# Novel (d)PCR assays for influenza A(H5Nx) viruses clade 2.3.4.4b surveillance

**DOI:** 10.2807/1560-7917.ES.2025.30.33.2500183

**Published:** 2025-08-21

**Authors:** Gerhard Buttinger, Mauro Petrillo, Viviana Valastro, Sabrina Marciano, Marika Crimaudo, Valeria D’Amico, Gabriele Leoni, Renaud Seigneuric, Valentina Paracchini, Piotr Robouch, Bénédicte Lambrecht, Bernd Manfred Gawlik, Calogero Terregino, Carolina Veneri, Giuseppina La Rosa, Elisabetta Suffredini, Maddalena Querci, Valentina Panzarin, Antonio Marchini

**Affiliations:** 1 European Commission, Joint Research Centre (JRC), Geel, Belgium; 2 Seidor Italy S.r.l., Milan, Italy; 3 EU/National Reference Laboratory for Avian Influenza and Newcastle Disease, Istituto Zooprofilattico Sperimentale delle Venezie (IZSVe), Legnaro, Italy; 4 European Commission, Joint Research Centre (JRC), Ispra, Italy; 5 Avian Virology and Immunology, Sciensano, Brussels, Belgium; 6 National Center for Water Safety (CeNSiA), Istituto Superiore di Sanità, Rome, Italy; 7 Department of Food Safety, Nutrition and Veterinary Public Health, Istituto Superiore di Sanità, Rome, Italy; * These authors contributed equally

**Keywords:** Avian influenza, HPAI A(H5N1) clade 2.3.4.4b, digital RT-PCR, qRT-PCR, wastewater surveillance, molecular diagnosis

## Abstract

**BACKGROUND:**

Since March 2024, cases of highly pathogenic avian influenza (HPAI) caused by A(H5N1) virus of clade 2.3.4.4b have been reported in dairy cattle in the United States, followed by spillover to avian and other mammalian species including humans. Although human-to-human transmission has not been reported, the virus's ability to infect mammals and potential of adaptation raise public health concerns, necessitating enhanced monitoring and preparedness.

**AIM:**

We aimed to develop digital RT-PCR assays to detect and quantify influenza A(H5N1) 2.3.4.4b viruses in biological and environmental samples.

**METHODS:**

We developed two digital RT-PCR assays targeting the matrix protein (JRC-MP) and haemagglutinin (JRC-HA) genes of A(H5N1) 2.3.4.4b viruses. After in silico assessment of inclusivity and exclusivity, we evaluated the assays’ performance using RNAs from influenza A(H5N1) viruses isolated from infected animal specimens, in an inter-laboratory exercise with diverse target and non-target isolates, and on wastewater samples either negative or spiked with A(H5N1) 2.3.4.4b RNA.

**RESULTS:**

The JRC-MP assay detects influenza A viruses of different subtypes and origins, while the JRC-HA assay specifically detects HPAI A(H5Nx) 2.3.4.4b strains. The assays demonstrated high sensitivity, showing consistent results in the inter-laboratory exercise. They also detected target RNAs in wastewater samples with high accuracy, despite background components, supporting potential use in wastewater surveillance programmes.

**CONCLUSIONS:**

Aligned with One Health strategies for zoonotic avian influenza surveillance, we propose the combined use of these two assays for the rapid and sensitive detection of influenza A(H5Nx) 2.3.4.4b in biological and environmental samples to enhance monitoring and outbreak control measures.

**Key public health message**

**What did you want to address in this study and why?**

We wanted to address the growing concern of highly pathogenic avian influenza (HPAI) A(H5N1) virus transmission between animals and from animals to humans. To provide effective tools to quickly detect and quantify recent avian influenza HPAI A(H5N1) 2.3.4.4b in biological and environmental samples, including those detected in milk cows in the United States, we developed two digital RT-PCR.

**What have we learnt from this study?**

Our digital RT-PCR assays can distinguish the HPAI A(H5N1) 2.3.4.4b virus from other A viruses, including seasonal human influenza, in a single test. These assays also work well on wastewater samples, making them suitable for disease surveillance programmes, including wastewater surveillance. This means we now have a reliable tool to track the spread of the virus in animal and human populations, and at their interface.

**What are the implications of your findings for public health?**

Our findings have implications for public health, enabling timely detection and quantification of HPAI A(H5Nx) 2.3.4.4b viruses. This allows for swift implementation of control measures to prevent virus spread and protect public health. Our assays can help mitigate the risk of avian influenza transmission between animals and from animals to humans, while mitigating the economic burden associated to this infection.

## Introduction

Avian influenza is a highly contagious respiratory viral infection of birds caused by influenza A viruses (AIVs). Most AIVs are designated as low pathogenicity viruses and are maintained by wild waterfowl and sea birds. Only AIVs of the H5 and H7 subtypes have shown the capability to produce highly pathogenic avian influenza (HPAI) causing severe multi-organ systemic infection associated to high morbidity and mortality rates in poultry [1]. Since the early 2000s, HPAI A(H5Nx) viruses of the goose/Guangdong lineage (gs/GD) have not only affected domestic poultry, but also wild birds, which have facilitated the intercontinental spread of that lineage through migratory routes. This contributed to the constant evolution of these viruses through re-assortment, resulting in the emergence of various genetic clades and subclades [1,2]. Globally, HPAI A(H5Nx) viruses have caused the death of more than 633 million poultry, including both direct mortality from infection and indirect losses due to culling, and a sharp increase in the number of affected wild birds and mammalian species, highlighting their impact on the food industry and biodiversity preservation [3]. 

Since March 2024, numerous cases of HPAI caused by A(H5N1) virus of clade 2.3.4.4b have been reported in the United States (US) also in dairy cattle, with more than 1,050 confirmed cases across 17 states as of June 2025. The virus has also spilled back to birds and transmitted to other mammalian species, including domestic cats. Since April 2024, there have been 70 confirmed human cases in the US, mostly related to exposure to sick or infected dairy cows and a smaller number linked to infected poultry [4]. While most human cases have been mild, with symptoms limited to conjunctivitis and minor respiratory issues, two severe cases, both referable to genotype D1.1, were reported: a person in their 60s with fatal outcome, and a teenage patient who recovered after hospitalisation [5-7]. 

Although human-to-human transmission has not been reported, the continuous evolution and adaptation of HPAI A(H5Nx) 2.3.4.4b strains and their potential for genetic re-assortment with seasonal or other mammalian influenza viruses is a concern for public health [2]. Recent studies have shown that a single mutation in the HA protein (i.e. Q226L substitution) was sufficient to change the receptor-binding preference from avian-type to human-type receptors, leading to increased risk for human-to-human transmission [8]. Further genetic analysis of the viruses from the two patients with severe disease revealed additional mutations in the HA protein associated with increased ability to bind to human cells present in the upper airways [7], suggesting that the virus may rapidly acquire new changes in its viral genome that could facilitate human infection [9]. Continued monitoring and surveillance of emerging mutations are important, as is the development of effective tools to accelerate diagnosis. This includes surveillance in livestock, wild, feral and companion animals, and exposed humans, as well as environmental monitoring in both natural and urban contexts [10].

Wastewater surveillance can indeed provide rapid and reliable information on virus circulation in a given area or period regardless of access to medical testing [11]. The real-time reverse transcription PCR (qRT-PCR) assay is the first-choice laboratory test for fast and accurate detection and quantification of influenza virus [11], and a number of H5 subtyping/pathotyping assays are available for use in veterinary and clinical contexts [12-14]. However, their application to wastewater requires optimisation owing to the complexity of the sample matrix composition that can affect test performance. The digital RT-PCR (dRT-PCR) technology offers greater resilience to PCR inhibitors and allows viral genome quantification with high sensitivity, without the need for calibration curves [15]. Accurate quantification of viral genome copies allows also estimating the number of infected individuals in an area and reconstructing infection trends in a population. Application of dRT-PCR to wastewater surveillance in the US has detected H5 viral RNA in several states, most probably introduced through industrial discharge of animal waste from poultry farming and/or milk by-products [16-18]. However, the applied protocols broadly react with H5 viruses and do not distinguish A(H5Nx) 2.3.4.4b viruses [17].

Building on our previous experience with in silico design and validation of dRT-PCR assays for severe acute respiratory syndrome coronavirus 2 (SARS-CoV-2) [11,19,20], this study aimed to develop a specific dRT-PCR assay to monitor the widespread and cross-species circulation of A(H5N1) clade 2.3.4.4b, while preparing for potential shifts in the epidemiological landscape in Europe. 

## Methods

### In silico assay design

The assays were designed by applying a bioinformatics workflow previously established for SARS-CoV-2 [20], which was specifically adapted to target ultra-conserved elements within the HA and M genetic segments of HPAI A(H5N1) viruses of clade 2.3.4.4b encoding for the haemagglutinin (HA) protein and matrix proteins (MP) M1 and M2, respectively. The workflow implements, for each of the two segments, the procedure described below:

1. We selected sequences annotated as ‘complete segment’ of HPAI A(H5N1) viruses of clade 2.3.4.4b, deposited in GISAID EpiFlu database [21] from 1 January 2022 until 30 May 2024, resulting in 12,783 submitted isolates from animals worldwide. Of these, we considered only isolates in which both the HA and M segments were marked as fully sequenced, for a total of 9,700 complete sequences for each segment.
2. Sequences were extracted and subjected to clustering analysis by using *cd-hit* tool as described in [20] to identify groups of closely related sequences applying an identity threshold of 0.98.
3. Sequences of the largest identified cluster (composed of 6,214 and 6,762 sequences for the HA and the M segment, respectively) were aligned by using the *mafft* tool as described in [20]. The presence of sequences related to the 2024 US outbreak viruses was confirmed in the largest cluster to ensure the procedure’s applicability for monitoring this outbreak of concern.
4. We used the alignment for the generation of a consensus sequence with the same threshold reported in [20].
5. We used the consensus sequence to design sets of primers and probes. The initial selections recommended by the primer design tool Primer3Plus [22] were adopted as the primary choices for the assays’ optimisation.

### In silico analysis

Cross-reactivity was assessed by aligning the primers and probe sequences against publicly available sequences from the entire NCBI database (with a focus on the *metazoa* kingdom, taxid:33208) using the *blastn* tool with very relaxed parameters (i.e. *blastn* with word size of seven bases and no low-complexity filtering).

All in silico PCR simulations were run with the thermonucleotideBLAST software version 2.17 as previously outlined [20]. Unless otherwise specified, we used default parameters, with the following specifications: minimum primer Tm (-e) = 40, minimum probe Tm (-E) = 40, maximum number of allowed mismatches (--max-mismatch) = 5 and maximum amplicon length (-l) = 200. Each simulation and its subsequent assessment were conducted independently.

True positives and false negatives were evaluated by simulating PCR on the initial set of 9,700 HPAI A(H5N1) RNA sequences. For the true positives, in silico PCR was run with stringent conditions i.e. allowing maximum one mismatch in the entire oligonucleotide set upstream the last five bases of the primer 3’-end (--primer-clamp = 5). The number of putative false negatives was determined by counting sequences which had at least one mutation in the last five bases of the primer 3’-end regions [23].

To further assess in silico the assays’ specificity for avian influenza A viruses circulating worldwide in the last year, we performed PCR simulation against all avian influenza A virus sequences (encompassing all subtypes, both high- and low-pathogenicity strains that had been detected, irrespective of their host species of origin) deposited in GISAID from January to December 2024 (n = 35,838), i.e. a set of sequences which provided a comprehensive picture of current influenza A viruses. To verify that the assays would remain effective in detecting emerging highly pathogenic clades, we repeated the analysis using a dataset of HPAI and low pathogenic avian influenza (LPAI) A(H5Nx) isolates, that had recently circulated worldwide and were collected in GISAID between 1 January 2024 and 16 June 2025. These include 9,253 and 73 isolates labelled as HPAI and LPAI, respectively.

### Optimisation of JRC-MP and JRC-HA assays 

Optimisation of the JRC-MP and JRC-HA assays was carried out on a QX600 Droplet Digital (dd) PCR System (Bio-Rad) employing a one-step ddRT-PCR Advanced Kit for Probes (Bio-Rad). The primers and probes were used at the concentration of 900 and 400 nM, respectively. The JRC-MP and JRC-HA assays were performed in a simplex format unless otherwise specified. A 5.5 µL RNA sample was then added to 16.5 µL of reaction mix. Droplet generation of PCR reaction mixtures was performed using AutoDG (Bio‐Rad) with DG32 cartridge (Bio‐Rad). The HPAI A(H5N1) RNA, extracted from A/Vulpes Vulpes/Belgium/04016_0001/2023 isolate was used for the annealing temperature optimisation and LOD calculation by digital droplet RT-PCR (ddRT-PCR). Background details on this isolate are appended in Supplementary Table S1. 

To optimise the annealing temperature, we ran a temperature gradient from 50 to 62 °C in eight steps using the C1000 Touch Thermal Cycler (Bio‐Rad). The optimal annealing temperature (60 °C) was selected based on separation of positive and negative droplet clusters as well as minimal rain (ambiguous droplets). Positive and negative partitions were quantified with the QX600 droplet reader and the QXManager (v. 2.2.0.71) using an average droplet volume of 0.797 nL, as determined by microscopy. Reactions with fewer than 10,000 droplets were discarded from downstream analysis.

To assess the analytical sensitivity of the assays expressed as limit of detection (LOD), the RNA sample was serially diluted with Ambion carrier RNA (10 ng/µL) to obtain eight concentrations ranging from 20 to 0.2 copies per µL. Each dilution was measured 10 times. The LOD for both assays was calculated by applying an exponential model according to the equation: Y = Y_0_ + (Y_1_ − Y_0_) × (1 − exp(−K × x)) using a threshold of 95% [24].

We evaluated the JRC-HA and JRC-MP assays by RT-dPCR analysis of influenza A and influenza B virus RNAs, and compared their performance with the assay established by the US Centers for Disease Control and Prevention (CDC) and published in 2022 [25] that targets the matrix gene segment of the influenza A virus and the non-structural gene segment of the influenza B virus. As templates, we used the Twist Respiratory Virus Controls (Twist Bioscience), specifically the Synthetic Influenza H1N1 (2009) RNA Control for influenza A virus and the Synthetic Influenza B RNA Control for influenza B virus, as previously described [26].

Ultimately, to assess the functionality of the newly developed primers and probes in a reverse transcription-quantitative PCR (RT-qPCR) format, amplification reactions were carried out using a TaqPathTM 1 Step RT-qPCR Master Mix CG (Applied Biosystems) on a Quantstudio 7 Flex instrument (Applied Biosystems). Oligonucleotide concentrations were the same as for the ddPCR assays.

### Assay validation

We first evaluated the two assays using the RNA extracted from five isolates from different animal species positive for HPAI A(H5N1), provided by the National Reference Laboratory for Avian Influenza hosted by Scientific Direction of Animal Infectious Diseases at Sciensano. For details on the isolates we refer to Supplementary Table S1. The five samples were tested using the JRC-HA and JRC-MP assays in both simplex and duplex formats for comparison.

We then assessed reproducibility of the methods in an inter-laboratory comparison study using a panel of 40 samples provided by the EU and National Reference Laboratory for Avian Influenza and Newcastle Disease hosted by the Istituto Zooprofilattico Sperimentale delle Venezie (IZSVe). A list of these samples is appended in Supplementary Table S2. All viruses were grown in the allantoic cavity of specific pathogen-free (SPF) embryonated chicken eggs and were originally isolated from different bird species, except for two isolates from swine. Samples of avian origin consisted of 26 A(H5N1) 2.3.4.4b isolates, three A(H5Nx) LPAI strains and eight A(non-H5) strains. Isolates were first tested by qRT-PCR [27,28] to infer their load based on the quantification cycle (Cq) value. Afterwards, they were diluted in an inactivating guanidine thiocyanate-based commercial medium (PrimeStore MTM, Longhorn Vaccines and Diagnostics) so as to obtain a wide concentration range of target and non-target RNAs. Specifically, high-concentration H5 and non-H5 LPAI strains were used to verify the absence of cross-reactivity. We used H5 HPAI strains of clade 2.3.4.4b at various concentrations to operate within a wider dynamic range that simulated diverse contamination scenarios. The panel also included three AIV-negative allantoic fluids harvested from uninfected SPF embryonated chicken eggs and diluted in the same buffer. 

Double-anonymised testing was conducted at the European Commission’s Joint Research Centre (JRC) and at the IZSVe laboratories. The RNA extraction was performed using the QIAamp Viral RNA kit (QiAGEN). Two parallel RNA extractions were carried out for each sample, and the recovered RNA was pooled, yielding a total volume of 120 µL. The JRC-MP and JRC-HA assays were performed as simplex reactions in triplicate in each laboratory. The identity of the samples was disclosed after the tests were completed. 

### Digital RT-PCR analyses on wastewater samples

We evaluated the two assays on a panel of wastewater samples, including 10 SARS-CoV-2-positive samples, eight samples positive for human influenza A or B, and seven samples negative for the three viruses as assessed by the Istituto Superiore di Sanità in the context of the Italian environmental surveillance activities in urban wastewater. These wastewater samples were collected across Italy from November 2022 to April 2024. The sampling methodology, viral concentration and nucleic acid isolation have been previously described [11,29]. This panel was intended to demonstrate the applicability of the JRC-MP and JRC-HA methods on wastewater samples. 

The RNA extracted from wastewater samples was analysed with the QIAcuity Four 5-plex digital PCR system (Qiagen) using the 4x One-Step Advanced Probe Master Mix (Qiagen) according to the manufacturer’s instructions. Primers were used at 900 nM and probes at 200 nM (JRC-HA) or 100 nM (JRC-MP). For these samples, the JRC-HA and JRC-MP assays were run in a duplex format, in a single replicate due to limited availability of RNA sample. The same analysis was repeated after spiking in influenza A(H5N1) 2.3.4.4b RNA into the samples (ca 400 copies per reaction). The wastewater samples were also tested with the JRC-CoV-UCE.2 assay, specific for SARS-CoV-2 [20] and the CDC human Influenza A and B duplex assay [26]. Thermal cycling was conducted with an annealing temperature of 60 °C (JRC assays) or 55 °C (CDC assay). We used 24-well nanoplates with 26,000 partitions. Partitions were imaged with 500 ms exposure time and a gain setting of 6 for both the green and yellow channels. The QIAcuity Software Suite version 2.5.0.0 was used to determine sample thresholds according to the manual global threshold approach.

### Statistical analysis

We applied Youden plots to demonstrate the equivalence of (i) the duplex vs simplex assays and (ii) the results provided by the JRC and IZSVe laboratories. To further validate inter-laboratory reproducibility, we constructed the Bland–Altman plot (ratio vs average) [30] and a log_10_ relative standard deviation (RSD) vs log_10_ mass fraction plot for all 40 samples, as suggested by Thompson [31] and Côté [32]. All graphs were generated using GraphPad (version 10.0.0 for Windows, GraphPad Software, US).

## Results

### Methods design and in silico assessment

We designed the primers and probes for the JRC-HA and JRC-MP assays targeting conserved regions of HA and M viral genomic segments of HPAI A(H5N1) 2.3.4.4b (Table 1).

**Table 1:** Design of two PCR assays for detection of influenza A(H5Nx) clade 2.3.4.4b

Code	Oligonucleotide	Length (bp)	GC (%)	Tm (°C)	Amplicon size
JRC-HA					
HA01-Fwd	5’-ACTGGGCTCAGAAATAGTCCTC-3’	22	50.0	59.2	139 bp
HA01-Rev	5’-TCCCCTGCTCATTGCTATGATG-3’	22	50.0	60.2	
HA01-Prb	5’-FAM-AGAGGGAGGATGGCAGGGAATGGTT-QSY7–3’	25	56.0	60.9	
Amplicon	ACTGGGCTCAGAAATAGTCCTCTAAGAGAAAAGAGAAGAAAAAGAGGTCTGTTTGGGGCGATAGCAGGGTTTATAGAGGGAGGATGGCAGGGAATGGTTGATGGTTGGTATGGGTACCATCATAGCAATGAGCAGGGGA				
JRC-MP					
MP01-Fwd	5’-TGTCTTTGCAGGGAAGAACACC-3’	22	50.0	61.0	114 bp
MP01-Rev	5’-ACGGTGAGCGTGAACACAAATC-3’	22	50.0	61.9	
MP01-Prb	5’-VIC-ATCTTGAGGCTCTCATGGAATGGCT-QSY7–3’	25	48.0	57.8	
Amplicon	TGTCTTTGCAGGGAAGAACACCGATCTTGAGGCTCTCATGGAATGGCTAAAGACAAGACCAATCCTGTCACCTCTGACTAAGGGGATTTTGGGATTTGTGTTCACGCTCACCGT				

Fwd: forward primer; JRC: Joint Research Centre; Prb: probe; Rev: reverse primer; Tm: melting temperature.

For each assay, the reported amplicon is the most frequently observed sequence targeted by the assay in the United States outbreak sequence segments deposited in GISAID (December 2024).

In silico PCR simulations on the initial set of 9,700 influenza A(H5N1) clade 2.3.4.4b viral sequences predicted that 9,237 (95.2%) and 9,274 (95.6%) sequences were detectable under stringent conditions (maximum five mismatches) by the JRC-HA and JRC-MP assays, respectively. Both assays were extensively tested in silico by sequence similarity searches for potential cross-reactivity with animal and human genomes found in publicly available databases. No significant similarity was observed between target regions of HA and M segments and non-target genomes.

**Figure 1:**
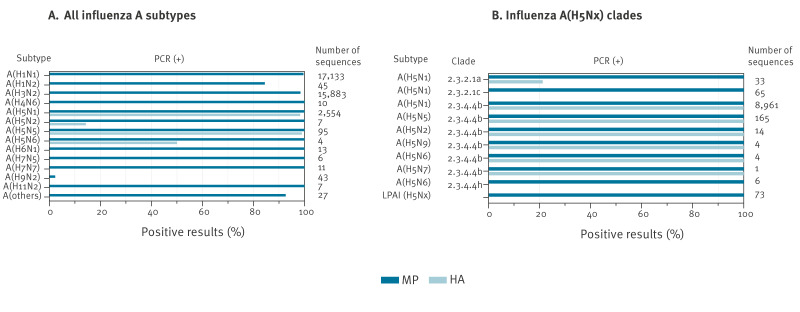
In silico PCR simulations for two assays targeting the haemagglutinin and matrix genes of influenza A(H5Nx) clade 2.3.4.4b

HA: JRC-HA assay; HPAI: highly pathogenic avian influenza; JRC: Joint Research Centre; LPAI: low pathogenic avian influenza; MP: JRC-MP assay.

We carried out further analysis against a set of 35,838 avian influenza A virus sequences deposited in GISAID in 2024, including high and low pathogenicity subtypes. The JRC-HA assay showed high specificity for HA segments from HPAI A(H5Nx) isolates of clade 2.3.4.4b, detecting 98.3% and 98.9% of A(H5N1) and A(H5N5) sequences, respectively, with only 44 sequences failing detection (Figure 1A). In contrast, no in silico amplification was observed for HA segments from other subtypes, even under relaxed conditions (up to 10 mismatches allowed across the oligonucleotides’ hybridisation region). The JRC-MP assay showed a lack of specificity, as it was predicted to amplify M sequences from multiple subtypes of influenza A viruses (Figure 1A). This is consistent with the high conservation of the M segment among different influenza A subtypes.

The x-axis represents the percentage of successful in silico PCR amplifications. Samples in panel A: influenza A virus subtypes circulating in 2024 and deposited in GISAID. Subtypes with fewer than four sequences available in GISAID are grouped under category ‘others’ (detailed in Supplementary Table S3). Samples in panel B: all HPAI and LPAI A(H5Nx) subtypes deposited in GISAID from 1 January 2024 to 16 June 2025 and labelled as HPAI (including clade designation) and LPAI, respectively.

To further evaluate the assays' ability to detect currently circulating high pathogenicity clades, we repeated the in silico PCR simulations using a dataset including all worldwide HPAI (n = 9,253) and LPAI (n = 73) A(H5Nx) sequence isolates collected between 1 January 2024 to 16 June 2025 and deposited in GISAID. While the LPAI sequences were detected only by the JRC-MP and not by the JRC-HA assay, the HPAI sequences were recognised by both the JRC-MP and JRC-HA assays with an in silico detection rate of 100% and 98.9%, respectively (Figure 1B).

At clade level, the JRC-HA assay in silico detected almost all 2.3.4.4b viruses (n = 9,849) from different subtypes with only three A(H5N1) sequences missed which had HA genes that were either incomplete (two sequences) or of low quality (one sequence with ambiguous nucleotides in the target region). Clade 2.3.2.1a was partially detected with seven of 26 sequences yielding positive results, while clade 2.3.2.1c (n = 65) and clade 2.3.4.4h (n = 6) isolates gave negative results. Detection was restored when applying more relaxed parameters (i.e. allowing a maximum of six mismatches).

### Assay optimisation

**Figure 2:**
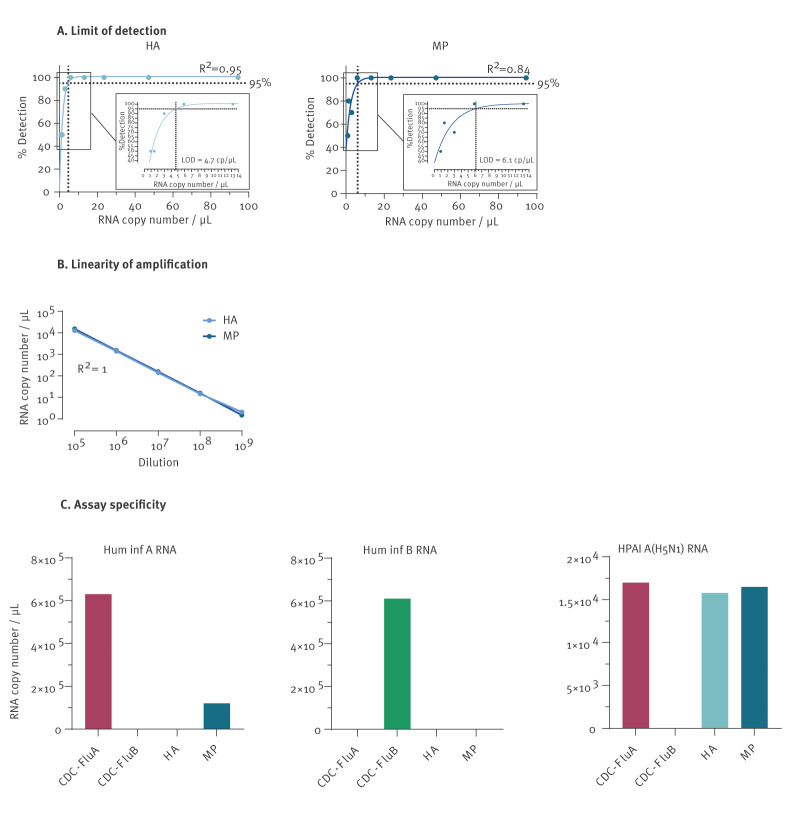
Performance evaluation of two digital PCR assays for the detection of influenza A(H5Nx) clade 2.3.4.4b

CDC: United States Centers for Disease Control and Prevention; Cp: copies; dd: droplet digital; HA: JRC-HA assay; HPAI: highly pathogenic avian influenza; Hum inf: human influenza; JRC: Joint Research Centre; LOD: limit of detection; MP: JRC-MP assay.

We first carried out annealing temperature optimisation for the JRC-HA and JRC-MP, and the temperature of 60 °C was selected as optimal for both assays, allowing clear discrimination of positive and negative partitions. We determined LOD values of 4.7 and 6.1 RNA copies per µL for the JRC-HA and the JRC-MP assays, respectively, with R^2^ values of respectively 0.95 and 0.84 (Figure 2A).

Panel A: LOD were determined by ddRT-PCR analysing a HPAI A(H5N1) 2.3.4.4b positive RNA isolated from infected *Vulpes vulpes*. Panel C: Synthetic control RNAs for human influenza A, human influenza B (Twist Bioscience) or HPAI A(H5N1) 2.3.4.4b RNA were used as a template. The performance of JRC-HA and JRC-MP assays was compared with the CDC human influenza duplex assay. Both JRC and CDC assays were run in a duplex format.

A linear response was observed for RNA concentrations ranging from 20 to 10,000 copies per reaction (Figure 2B). When tested on a RT-qPCR format, the assays yielded similar sensitivity, demonstrating the versatility and compatibility of the assays across different PCR formats. For details on the amplification plots limit of detection analysis see Supplementary Figure S1.

To further evaluate the specificity of the assay, in addition to HPAI A(H5N1) RNA we also used Twist synthetic RNA controls for human seasonal influenza A and influenza B viruses as templates for the dRT-PCR. The CDC assay specific for human seasonal influenza A and B viruses was used for comparison [25]. The JRC-HA assay exhibited high specificity, with no detectable signal from seasonal influenza A and B RNAs. In contrast, the JRC-MP assay cross-reacted with human influenza A virus, although its sensitivity was approximately sixfold lower than that of the reference CDC assay (Figure 2C). Notably, the CDC assay for seasonal influenza virus detected HPAI A(H5N1) RNA with a sensitivity similar to the JRC-MP and JRC-HA assays.

### Analysis on avian influenza A isolates

**Figure 3:**
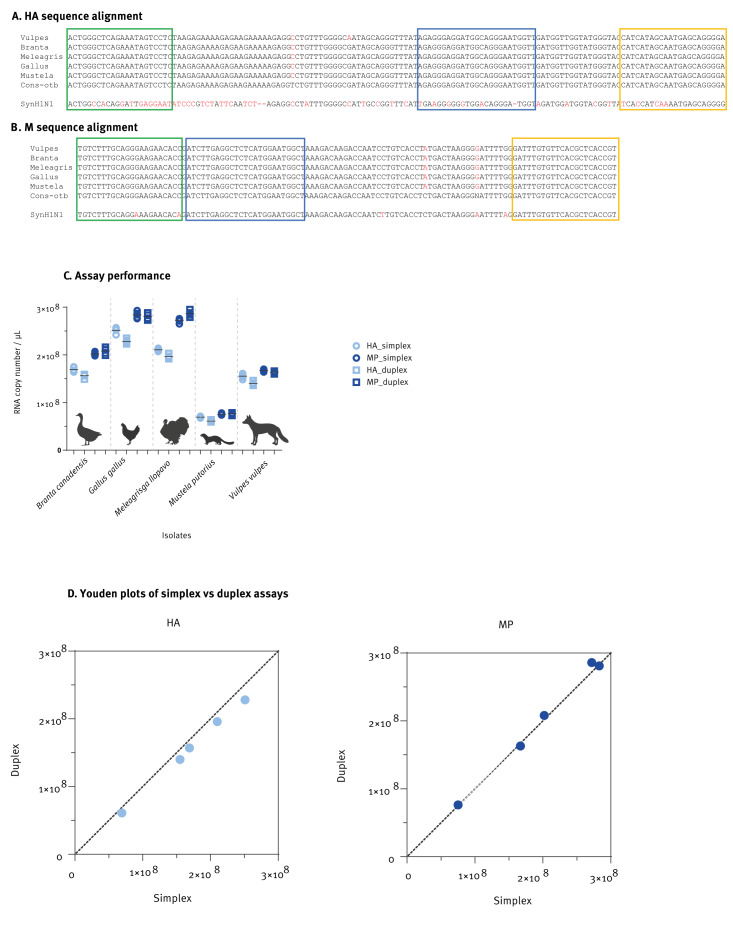
Validation of two digital PCR assays for the detection of influenza A(H5Nx) clade 2.3.4.4b, Belgium, 2022–2023 (n = 5)

CDC: United States Centers for Disease Control and Prevention; dd: droplet digital; HA: JRC-HA assay; HPAI: highly pathogenic avian influenza; JRC: Joint Research Centre; MP: JRC-MP assay.

To confirm the performance of the JRC-HA and JRC-MP assays, we selected four additional HPAI A(H5N1)-positive isolates. The characteristics of these samples are described in Supplementary Table S1. The primer and probe sequences of the JRC-HA and JRC-MP assays showed 100% identity to their respective binding sites on the viral genome prototype, as defined by alignment of the first 21 sequence segments of the 2024 US outbreak. The same target regions were also present in the isolates we selected for the analysis (Figure 3A).

Panels A and B: PCR target sequence segments of the selected isolates (details listed in Supplementary Table S1) are aligned with the corresponding regions of consensus sequences (cons-otb) generated by analysing the first 21 sequences (from goats, cows, cats and humans) of the United States outbreak deposited in GISAID by the CDC. The sequences of the synthetic human influenza A control material used in Figure 2 (labelled as SynH1N1) are also included. The forward, probe and reverse target regions are boxed in green, blue and yellow, respectively. Bases different from those of the consensus are highlighted in red. Panel C: Five different RNAs were analysed by ddRT-PCR in five replicates by ddRT-PCR using the JRC-HA and JRC-MP assays performed in both simplex and duplex formats. Black bars indicate the average.

The JRC-HA and JRC-MP assays were performed singly or as a duplex assay. Both assays successfully detected the viral target sequences with comparable performance (Figure 3C). The Youden plots (Figure 3D) indicate that the simplex and duplex assay formats yielded similar results.

A second panel of 40 samples, comprising high- and low-pathogenic type A influenza viruses isolated from different bird species and swine, as well as three negative controls, was analysed in parallel at the JRC and at the IZSVe in an inter-laboratory reproducibility study. Details of these samples can be found in Supplementary Table S2. Qualitative results between the two laboratories were in perfect agreement for all the samples (Table 2).

**Table 2:** Qualitative inter-laboratory analysis of an anonymised panel of influenza samples using the JRC-HA and JRC-MP digital PCR assays (n = 40)

Subtype	Pathotype	Lineage/clade	HA IZSVe +	HA JRC +	% HA agreement	MP IZSVe +	MP JRC +	% MP agreement
H1N1	LPAI	Avian	0/1	0/1	100	1/1	1/1	100
H1N1	LPAI	Swine 1A.3.3.2 [37]	0/1	0/1	100	1/1	1/1	100
H3N2	LPAI	Swine	0/1	0/1	100	1/1	1/1	100
H3N8	LPAI	Eurasian	0/2	0/2	100	2/2	2/2	100
H5N1	HPAI	Eurasian, 2.3.4.4b	26/26	26/26	100	26/26	26/26	100
H5N1	LPAI	Eurasian	0/1	0/1	100	1/1	1/1	100
H5N2	LPAI	Eurasian	0/1	0/1	100	1/1	1/1	100
H5N3	LPAI	Eurasian	0/1	0/1	100	1/1	1/1	100
H7N7	HPAI	Eurasian	0/1	0/1	100	1/1	1/1	100
H7N7	LPAI	Eurasian	0/1	0/1	100	1/1	1/1	100
H9N2	LPAI	Eurasian, clade Y8 [38]	0/1	0/1	100	1/1	1/1	100
Allantoic fluid			0/3	0/3	100	0/3	0/3	100
**Total**			**40**	**40**	**100 **	**40**	**40**	**100**

+: only the positive results are reported; HA: JRC-HA assay ; HPAI: high pathogenic avian influenza virus; IZSVe: Istituto Zooprofilattico Sperimentale delle Venezie; JRC: Joint Research Centre; LPAI: low pathogenic avian influenza virus; MP: JRC-MP assay.

The number of positive samples over the total number of isolates for a given subtype is shown. Unless otherwise specified, all samples were originally isolated from bird species.

In agreement with the in silico analyses, the JRC-HA assay was highly specific against HPAI A(H5N1) 2.3.4.4b, whereas the JRC-MP assay demonstrated a broader detection capability, identifying not only HPAI A(H5N1) 2.3.4.4b but also other Eurasian LPAI viruses of different subtypes (H1N1, H3N2, H3N8, H5N1, H5N2, H5N3, H7N7 and H9N2) as well as swine influenza strains.

**Figure 4:**
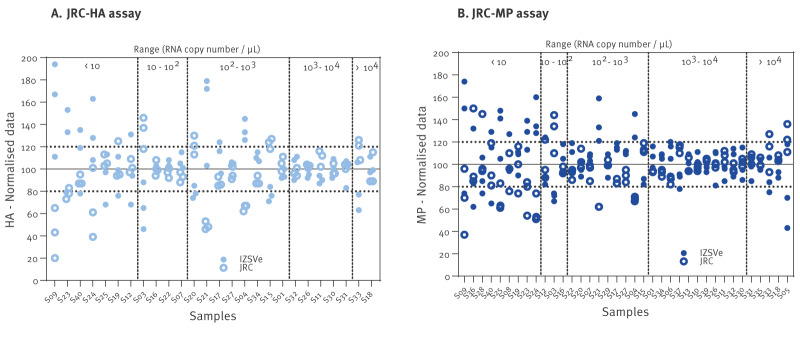
Quantitative inter-laboratory analysis of an anonymised panel of influenza samples using the JRC-HA and JRC-MP PCR assays (n = 40)

IZSVe: Istituto Zooprofilattico Sperimentale delle Venezie; JRC: Joint Research Centre; LPAI: low pathogenic avian influenza.

Quantitative analyses revealed a high degree of consistency between the two laboratories (Figure 4), which was also supported by statistical analyses, including Brand–Altmann and Youden plots; we provide these statistical results in Supplementary Figure S2. As expected, variability increased as the target concentration decreased, approaching the limit of quantification of the assays (Figure 4). Statistical analysis using Coté plots demonstrated strong agreement between the results obtained with the JRC-HA and JRC-MP assays with only minor discrepancies; the individual plots can be accessed in Supplementary Figure S2.

Samples were analysed in triplicate at the JRC and the IZSVe. To facilitate comparison, the six replicates for each sample were normalised by dividing them by their respective mean values. The data are arranged in ascending order based on their mean RNA copies/µL. The results are categorised into five distinct classes of concentration, demarcated by vertical blue dotted lines.

### Analysis on wastewater samples

**Figure 5:**
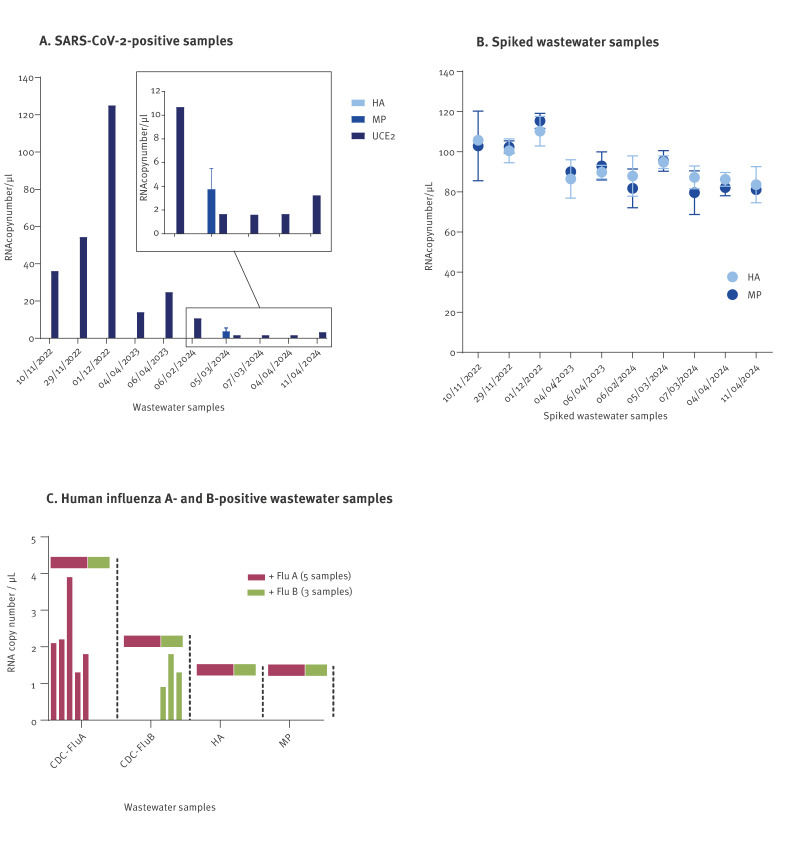
Evaluation of JRC-HA, JRC-MP PCR assays targeting influenza A(H5Nx) clade 2.3.4.4b in wastewater samples

HA: JRC-HA assay; MP: JRC-MP assay.

The testing results for wastewater specimens confirmed the presence of SARS-CoV-2 in all the samples as assessed by the JRC-CoV-UCE.2 assay [20]. The JRC-MP assay yielded positive results in one of the samples tested (March 2024), while the JRC-HA was negative in all samples (Figure 5A). After spiking the same samples with nominal 400 copies of HPAI A(H5N1) 2.3.4.4b RNA virus per reaction (corresponding to 100 copies/µL), the JRC-HA and JRC-MP assays maintained their specificity also in a background of wastewater RNA, yielding highly repeatable measurements (variation within 7%) (Figure 5B). To further evaluate the specificity of the assays in wastewater samples we also analysed eight wastewater samples positive for human influenza A or B along with seven influenza-negative samples. The JRC-HA and JRC-MP assays were run in parallel with the CDC duplex assay specific for seasonal influenza A and B viruses [25]. The CDC assay confirmed the presence of influenza viruses RNA influenza-positive samples as expected, whereas both the JRC-MP and JRC-HA assays yielded negative results (Figure 5C). All assays correctly identified the negative samples as negative (data not shown). These results confirm the high specificity of the JRC-HA assay that did not show any cross-reaction with seasonal influenza viruses. The negative results obtained with the JRC-MP assay were consistent with the relatively low levels of human influenza virus in the wastewater samples and the expected lower sensitivity of this assay compared with the CDC assay, as previously observed with synthetic RNAs (Figure 2C). This reduced sensitivity can be attributed to the presence of two mismatches in the annealing region of 3’-end of the forward primer, which may lead to suboptimal annealing to the target sequence within the M gene of human influenza A.

Panel A: Extracted RNA was analysed by chamber dRT-PCR using the JRC-HA, JRC-MP and JRC-UCE.2 assays, the latter specific for SARS-CoV-2. Samples are shown in a chronological order. Panel B: The same samples were analysed after addition of HPAI A(H5N1) 2.3.4.4b RNA virus. Dots show the mean values ± standard deviation. Panel C: RNAs extracted from wastewater samples and previously confirmed positive for Influenza A and B were analysed by dRT-PCR using the CDC duplex assay specific for human influenza A and B, and the JRC-HA and JRC-MP assays run in a duplex format.

## Discussion

The US is experiencing an outbreak of HPAI A(H5N1) clade 2.3.4.4b in dairy cows with incidental spill-over to other species including humans, sparking concerns about potential human-to-human transmission and severe clinical outcomes [8,9]. Controlling the virus’s evolution and closely monitoring its spread are critical, as timely interventions can mitigate the risk of larger outbreaks and protect public health. For this reason, wastewater surveillance has been undertaken in the US to assess trends in AIV distribution [16], and retail milk and dairy products are being tested [33,34]. Such screening relies on a broadly reactive H5 assay developed by Wolfe et al. [17]. While this method is valuable for initial assessment, the potential of cross-reaction with LPAI H5 strains due to the use of degenerated oligonucleotide requires downstream characterisation to ultimately confirm the presence of H5 2.3.4.4b genome.

Building on a successful approach used for SARS-CoV-2 [11,20], and grounding in the current epidemiological context that requires diagnostic precision and sensitivity, we developed two digital RT-PCR-based assays to detect HPAI A(H5Nx) viruses of clade 2.3.4.4b in biological and environmental samples. By leveraging ultra-conserved regions of the viral haemagglutinin gene segment, our JRC-HA assay demonstrates the ability to specifically detect the designated clade and offers a stable and reliable target for molecular diagnostics, with no cross-reactivity with LPAI subtypes. This is an agreement with other studies that have chosen the HA gene as a target for their assays [13]. As the N1 NA gene is a defining feature of these viruses, an additional assay developed for this target would provide more solid results in the current epidemiological framework. However, it must be noted that H5 viruses of clade 2.3.4.4b have shown a remarkable propensity to re-assort that resulted in a plethora of subtypes and genotypes [35]. As a consequence, the unpredictability of future evolutionary trajectories of 2.3.4.4b viruses discouraged us from using the N1 as a diagnostic target [36].

In contrast, the JRC-MP assay provides generic detection of influenza A viruses. Indeed, although oligonucleotides were designed using a dataset enriched for HPAI A(H5Nx) clade 2.3.4.4b sequences, the targeted ultra-conserved elements in the M gene are shared across different subtypes of HPAI and LPAI. Our panel validation showed that the assay has a broad detection capability for various influenza A viruses of avian and swine origin. In silico PCR analysis, performed under stringent conditions, further supported the assay's inclusivity, predicting that it could amplify most sequences across different subtypes. However, an exception were LPAI A(H9N2) sequences, which the JRC-MP assay may not amplify optimally due to mismatches between the 3’-end of primers and the targeted sequences. The potential for re-assortment between different influenza subtypes, including H5N1 and H9N2, highlights the importance of continued surveillance, informing assay updates that ensure the detection of emerging viruses. Interestingly, the JRC-MP assay exhibited reduced sensitivity compared with the qRT-PCR assay recommended by the CDC when applied to human influenza A synthetic RNA or in wastewater samples positive for seasonal influenza A. This reduced sensitivity is a deliberate design feature, as the JRC-MP assay was intentionally developed to prioritise detection of AIV while retaining limited cross-reactivity for human subtypes. Combined with the JRC-HA protocol, this multi-assay strategy enables accurate differentiation between avian HPAI A(H5Nx) 2.3.4.4b viruses and human influenza viruses, filling a surveillance gap by distinguishing avian from human signals in wastewater. 

It is important to note that while the JRC-MP and JRC-HA assays were validated in vitro using viral RNA extracted from different animals’ isolates, they were not evaluated on clinical samples from US cattle or from felines which have recently emerged as a relevant species highly susceptible to HPAI, nor did we evaluate them on milk samples. However, since the regions recognised by the oligonucleotide are identical between the American prototype sequences of clade 2.3.4.4b and the RNA samples used in this study, we can assume that the assays would perform efficiently both in the American and the European context. We further assessed in silico the applicability of the JRC-MP and JRC-HA protocols against clades other than 2.3.4.4b (i.e. 2.3.2.1a, 2.3.2.1c, 2.3.4.4h from Asia). A PCR simulation with more permissive conditions indicated that both assays can detect any of the currently circulating clades, although we might expect slightly lower sensitivity with the JRC-HA assay due to the presence of a higher number of mismatches in the annealing region. However, should the epidemiological situation change, our bioinformatics and laboratory pipeline allows updating and tuning the assay within 2 weeks, so that up-to-date diagnostic methods can be readily available.

While the assays are considerably more cost-effective than sequencing and we showed interchangeability between the dRT-PCR and standard qRT-PCR formats, their implementation as dRT-PCR requires investment in dedicated equipment or training for laboratories without existing infrastructure. However, their implementation in three different laboratories during the validation process support that the assays are robust and can easily be deployed to other facilities. 

Lessons from the COVID-19 pandemic emphasise the importance of rapid data sharing, including precise metadata such as date and location, to track fast-spreading pathogens. Gaps in metadata, as seen in avian influenza spillovers, hinder tracing and analyses. We faced challenges comparing data and ensuring quality due to missing standards, including reference sequences and the numbering of coding and amino acid sequences. Improved alignment with One Health strategies is needed. Harmonised sharing of assays, genomic data and workflows, as recommended by the European Food Safety Authority and the European Centre for Disease Prevention and Control, is essential to coordinated surveillance and prevention of further spillover events.

## Conclusions

The development of novel assays or detecting HPAI A(H5N1)/Nx) viruses of clade 2.3.4.4b represents a step forward in monitoring the potential spread of these viruses. These assays are designed to be highly adaptable, with applications spanning multiple sectors, including human health, animal health and environmental surveillance, and are well suited to support a comprehensive One Health approach.

## Data Availability

All the raw data supporting the findings of this work are available on request.

## Supplementary Data

543000 Supplement
