# Supplementary material for: Novel (d)PCR assays for influenza A(H5Nx) viruses clade 2.3.4.4b surveillance
Source: Euro Surveill. 2025 Aug 21;30(33):2500183. doi: 10.2807/1560-7917.ES.2025.30.33.2500183 (PMC12372895; doi:10.2807/1560-7917.ES.2025.30.33.2500183)
Supplement: Supplement [file 25-00183_MARCHINI_Supplement.pdf]

Supplementary material

This supplementary material is hosted by *Eurosurveillance* as supporting information alongside the article "Novel (d)PCR assays for Influenza A(H5Nx) viruses clade 2.3.4.4b surveillance", on behalf of the authors, who remain responsible for the accuracy and appropriateness of the content. The same standards for ethics, copyright, attributions and permissions as for the article apply. Supplements are not edited by *Eurosurveillance* and the journal is not responsible for the maintenance of any links or email addresses provided therein.

List of Supplementary materials

Supplementary Table 1. Details of the isolates used in Figure 2 and Figure 3.

Supplementary Table 2. Details of the sample panel used for assay validation in Figure 4, provided by IZSVe

Supplementary Table 3. Expanded list of influenza A virus subtypes categorised as 'Others' in Figure 1.

Supplementary Table 4. Characteristics of A(H5Nx) influenza virus isolates with *in silico* PCR detection failure.

Supplementary Figure S1: Performance of JRC-HA and JRC-MP assays on RT-qPCR platform.

Supplementary Figure S2: Inter-laboratory comparison: statistical analysis.

Supplementary Tables

Supplementary Table S1. Details of the isolates used for the assay validation. All isolates were kindly provided by Sciensano (Belgium). HPAI (subtype A(H5N1) clade 2.3.4.4b) samples were all collected from dead animals in various locations of Belgium.

Sample number	*Host*	*Collection Date*	*Host Status*	**GISAID Isolate Id**	**Isolate Name**
1	*Branta canadensis*	16/11/2022	Wild	EPI_ISL_18347728	A/Branta_canadensis/Belgium/13393_0002/2022
2	*Gallus gallus*	19/01/2023	Domestic	EPI_ISL_16809297	A/Gallus_gallus/Belgium/00548_0001/2023
3	*Meleagris gallopavo*	24/01/2023	Domestic	EPI_ISL_16809296	A/Meleagris_gallopavo/Belgium/00695_0001/2023
4	*Mustela putorius*	09/02/2023	Wild	EPI_ISL_17072114	A/Mustela_putorius/Belgium/01421_0001/2023
5	*Vulpes vulpes*	24/04/2023	Wild	EPI_ISL_17774570	A/Vulpes_Vulpes/Belgium/04016_0001/2023

Supplementary Table S2. Details of the sample panel used for the reproducibility study, provided by IZSVe (Italy).

Sample	Virus	Subtype	Pathotype	Lineage/Clade	Cq§
S01	A/buzzard/Italy/23VIR440-11/2023	H5N1	HPAI	Eurasian, 2.3.4.4b	29.04
S02	Negative				
S03	A/swan/Italy/23VIR123-15/2023	H5N1	HPAI	Eurasian, 2.3.4.4b	36.54
S04	A/turkey/Italy/23VIR10680-11/2023	H5N1	HPAI	Eurasian, 2.3.4.4b	30.09
S05	A/duck/Italy/21VIR8024-20/2021	H5N3	LPAI	Eurasian	23.37
S06	A/chicken/Italy/19VIR7171-48/2019	H3N8	LPAI	Eurasian	27.87
S07	A/chicken/Italy/22VIR10044-14/2022	H5N1	HPAI	Eurasian, 2.3.4.4b	32.07
S08	A/teal/Italy/22VIR4622-8/2022	H1N1	LPAI	avian	34.93
S09	A/swan/Italy/23VIR147-12/2022	H5N1	HPAI	Eurasian, 2.3.4.4b	36.75
S10	A/turkey/Italy/21VIR9117-15/2021	H5N1	LPAI	Eurasian	24.50
S11	A/turkey/Italy/23VIR1964-19/2023	H5N1	HPAI	Eurasian, 2.3.4.4b	26.46
S12	A/buzzard/Italy/23VIR440-11/2023	H5N1	HPAI	Eurasian, 2.3.4.4b	35.23
S13	A/mallard/Italy/22VIR4781-2/2022	H7N7	LPAI	Eurasian	23.55
S14	Negative				
S15	A/seagull/Italy/23VIR1310-12/2023	H5N1	HPAI	Eurasian, 2.3.4.4b	29.60
S16	A/seagull/Italy/23VIR1203-29/2023	H5N1	HPAI	Eurasian, 2.3.4.4b	33.74
S17	A/swan/Italy/23VIR883-13/2023	H5N1	HPAI	Eurasian, 2.3.4.4b	31.56
S18	A/chicken/Italy/23VIR3021-33/2023	H5N1	HPAI	Eurasian, 2.3.4.4b	24.38
S19	A/peregrine falcon/Italy/23VIR2255-11/2023	H5N1	HPAI	Eurasian, 2.3.4.4b	36.54
S20	A/chicken/Italy/23VIR3799-11/2023	H5N1	HPAI	Eurasian, 2.3.4.4b	32.41
S21	A/black-headed_gull/Italy/23VIR2983-11/2023	H5N1	HPAI	Eurasian, 2.3.4.4b	31.92
S22	A/buzzard/Italy/23VIR440-11/2023	H5N1	HPAI	Eurasian, 2.3.4.4b	32.47
S23	A/sandwich_tern/Italy/23VIR5828-17/2023	H5N1	HPAI	Eurasian, 2.3.4.4b	36.11
S24	A/mallard/Italy/22VIR9219-6/2022	H5N1	HPAI	Eurasian, 2.3.4.4b	35.63
S25	A/chicken/Italy/23VIR3021-33/2023	H5N1	HPAI	Eurasian, 2.3.4.4b	34.55
S26	A/swan/Italy/23VIR147-12/2022	H5N1	HPAI	Eurasian, 2.3.4.4b	26.29
S27	A/seagull/Italy/23VIR1203-29/2023	H5N1	HPAI	Eurasian, 2.3.4.4b	30.11
S28	A/teal/Italy/22VIR4622-10/2022	H9N2	LPAI	Eurasian, clade Y8*	25.32
S29	A/teal/Italy/22VIR8201-16/2022	H3N8	LPAI	Eurasian	30.31
S30	A/common crane/Italy/23VIR10524-12/2023	H5N1	HPAI	Eurasian, 2.3.4.4b	25.22
S31	A/sandwich_tern/Italy/23VIR5828-17/2023	H5N1	HPAI	Eurasian, 2.3.4.4b	25.26
S32	A/peregrine falcon/Italy/23VIR2255-11/2023	H5N1	HPAI	Eurasian, 2.3.4.4b	25.41
S33	A/seagull/Italy/23VIR1103-13/2023	H5N1	HPAI	Eurasian, 2.3.4.4b	24.86
S34	A/chicken/Italy/23VIR3022-26/2023	H5N1	HPAI	Eurasian, 2.3.4.4b	28.73
S35	A/pheasant/Italy/22VIR1741-97/2022	H5N2	LPAI	Eurasian	24.01
S36	A/swine/Italy/13VIR125-3/2013	H3N2	LPAI	Swine	30.10
S37	A/swine/Italy/13VIR6432-63/2013	H1N1	LPAI	Swine lineage 1A.3.3.2 - H1N1pdm09**	28.31
S38	Negative				
S39	A/chicken/Italy/16VIR1873-9/2016	H7N7	HPAI	Eurasian	26.10
S40	A/seagull/Italy/23VIR1203-29/2023	H5N1	HPAI	Eurasian, 2.3.4.4b	33.82

§Value obtained by qRT-PCR applying the assay developed by [1] as modified by [2]
* According to [3]
** According to [4]

References

[1] Heine HG, Foord AJ, Wang J, et al. Detection of highly pathogenic zoonotic influenza virus H5N6 by reverse-transcriptase quantitative polymerase chain reaction. Virol J. 2015;12:18.

[2] Laconi A, Fortin A, Bedendo G, et al. Detection of avian influenza virus: a comparative study of the in silico and in vitro performances of current RT-qPCR assays. Sci Rep. 2020;10(1):8441.

[3] Fusaro A, Pu J, Zhou Y, et al. Proposal for a Global Classification and Nomenclature System for A/H9 Influenza Viruses. Emerg Infect Dis. 2024;30(8):1-13.

[4] Anderson TK, Macken CA, Lewis NS, et al. A Phylogeny-Based Global Nomenclature System and Automated Annotation Tool for H1 Hemagglutinin Genes from Swine Influenza A Viruses. mSphere. 2016;1(6).

Supplementary Table S3. Expanded list of influenza A virus subtypes categorised as 'Others' in Figure 1A. The 'Others' category includes 27 GISAID entries from the analysed period. The number (N.) of entries retrieved and evaluated for each subtype is indicated. *In silico* PCR simulation shows that all entries are negative (-) for the HA assay, while most are positive (+) for the MP assay.

Subtype	N. of entries	HA	MP
A(H2N3)	2	-	+
A(H3N1)	2	-	+
A(H3N8)	1	-	+
A(H4N1)	3	-	+
A(H6N2)	2	-	+
A(H6N5)	1	-	+
A(H6N8)	1	-	+
A(H7N3)	1	-	+
A(H7N4)	1	-	+
A(H7N9)	1	-	+
A(H9N9)	1	-	+
A(H10N3)	1	-	-
A(H10N4)	1	-	+
A(H10N7)	2	-	+
A(H11N4)	1	-	+
A(H11N9)	2	-	+
A(H12N5)	2	-	+
A(H13N2)	1	-	-
A(H13N6)	1	-	+

Supplementary Figure S1

Performance of JRC-HA and JRC-MP assays on RT-qPCR platform

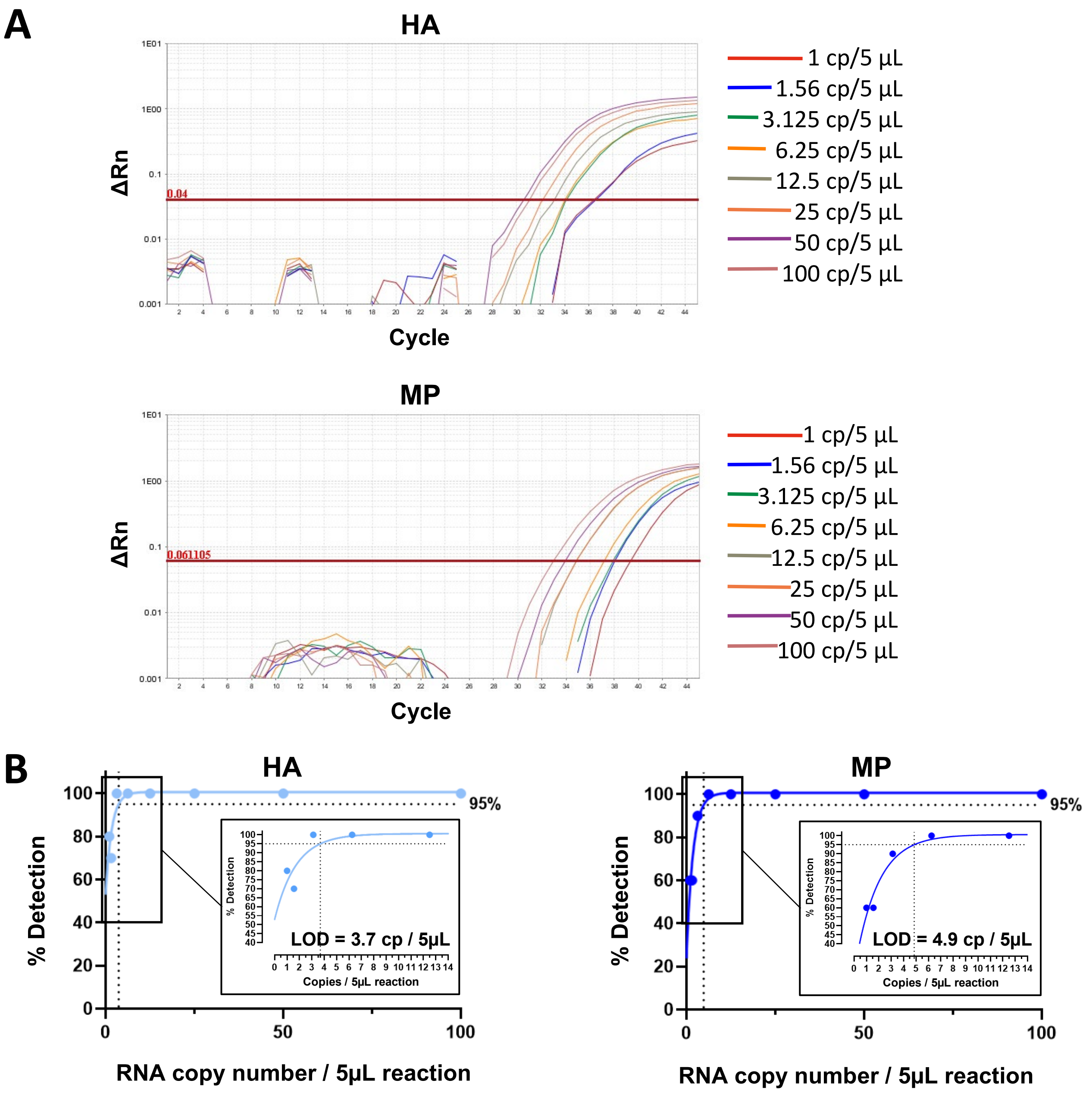

A. Amplification plots for the HA (upper panel) and MP (lower panel) assays using the HPAI A(H5N1) 2.3.4.4b positive RNA. **B.** Limit of detection (LOD) analysis for HA and MP assays. Serial dilutions of the RNA were prepared and analysed by qRT-PCR with the JRC-HA and JRC-MP assays, with each dilution tested in ten replicates. The x-axis represents the RNA concentration (copy number/5 µL reaction) (cp=copy number), and the y-axis shows the percentage of positive results. The LOD values, calculated as described in the Methods section, are indicated in the figure.

Supplementary Figure S2

Inter-laboratory comparison: statistical analysis.

A Bland-Altman plots

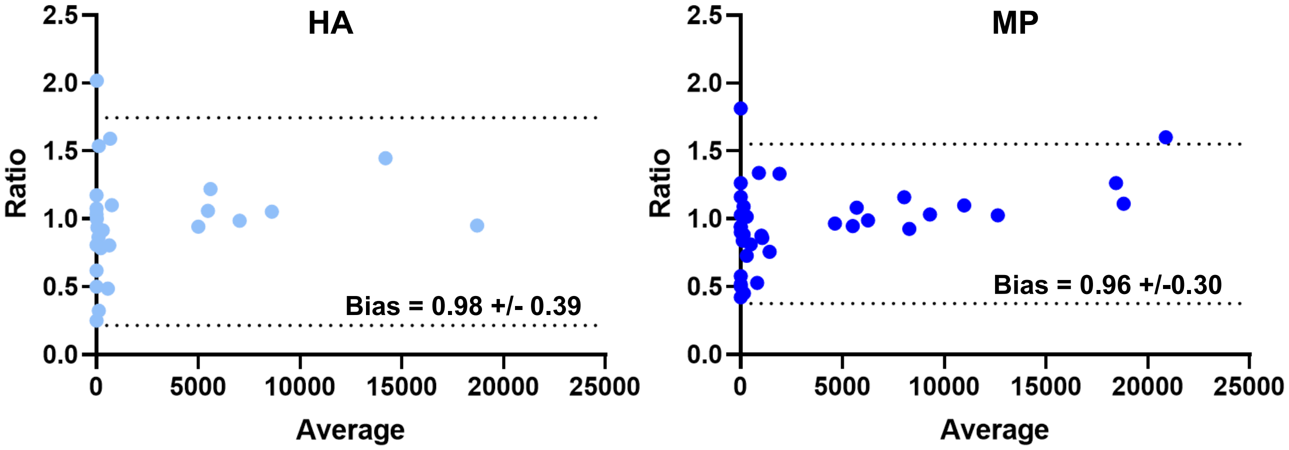

B Youden plots

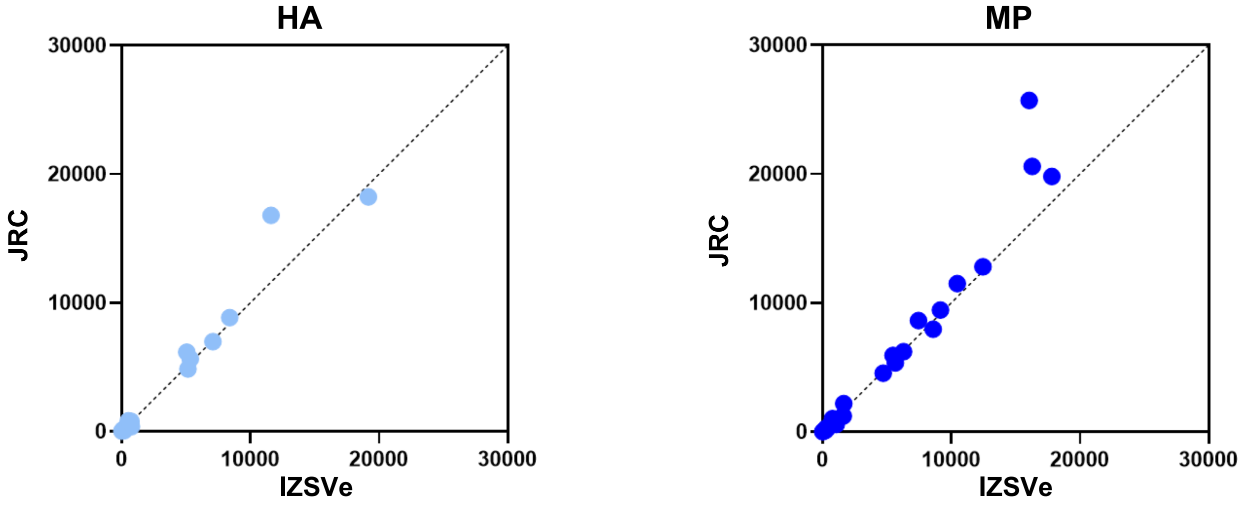

C

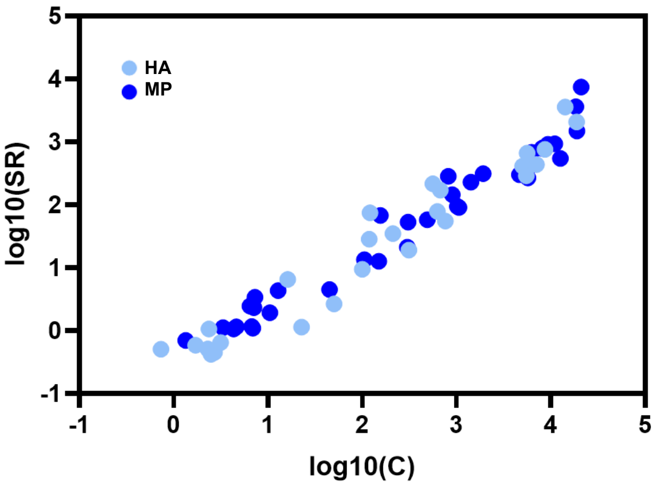

A. Bland-Altman (ratio vs average) plots for the HA (left panel, bias=0.98+/0.39) and the MP (right panel, bias=0.96+/0.30) assays. **B.** Youden plots for the HA (left panel, R^2=0.96) and the MP (right panel, R^2=0.96) assays for the JRC and the IZSVe. **C.** Côté plot (R^2=0.95) representing $\log_{10}$ of reproducibility standard deviation (S_R) as a function of $\log_{10}$ of grand average of the RNA sequence copy number/µL for HA and MP assays.
